# Supplementary material for: Hybrid Models and Biological Model Reduction with PyDSTool
Source: PLoS Comput Biol. 2012 Aug 9;8(8):e1002628. doi: 10.1371/journal.pcbi.1002628 (PMC3415397; doi:10.1371/journal.pcbi.1002628)
Supplement: Text S4 — Complete source code for the PyDSTool package (version 0.88.120504). Includes API documentation and help files linking to web pages. This file is identical to the current public release on Sourceforge.net. (ZIP) [file pcbi.1002628.s004.zip › PyDSTool/html/PyDSTool.Toolbox.optimizers.step.local_brute_force_1dstep-pysrc.html]

xml version="1.0" encoding="ascii"?


PyDSTool.Toolbox.optimizers.step.local\_brute\_force\_1dstep


| Home | Trees | Indices | Help | PyDSTool |
| --- | --- | --- | --- | --- |

Package PyDSTool ::
Package Toolbox ::
Package optimizers ::
Package step ::
Module local\_brute\_force\_1dstep

[hide private]

[frames] | no frames]

# Source Code for Module PyDSTool.Toolbox.optimizers.step.local\_brute\_force\_1dstep

```
 1  import numpy as np 
 2  from PyDSTool import common 
 3   


4 -class LocalBruteForce1DStep(object):


5      """Local brute force search for 1D parameter (sub-)space, making no 
 6      use of gradient information. Takes a "step" in a local neighbourhood to 
 7      the minimum in that neighbourhood. Specify the neighbourhood by absolute 
 8      limits as a pair or Interval object, and either the resolution (for uniform 
 9      sampling of the interval) or the explicit sample values (as a strictly 
10      increasing sequence), via keyword args 'resolution' or 'samples'. 
11   
12      Use in conjunction with SimpleLineSearch. 
13      """ 


14 -    def __init__(self, interval, **kwargs):


15          self.interval = interval 
16          if 'samples' in kwargs: 
17              self.samples = kwargs['samples'] 
18              assert self.samples[0] >= self.interval[0] 
19              assert self.samples[-1] <= self.interval[-1] 
20              assert common.isincreasing(self.samples) 
21          elif 'resolution' in kwargs: 
22              numpoints = 1 + (self.interval[1]-self.interval[0])/kwargs['resolution'] 
23              self.samples = np.linspace(self.interval[0], self.interval[1], 
24                                            numpoints) 
25          if 'smooth' in kwargs: 
26              self.smooth = kwargs['smooth'] 
27          else: 
28              self.smooth = True 
29          if 'index' in kwargs: 
30              self.index = kwargs['index'] 
31          else: 
32              self.index = 0 
33          if self.smooth: 
34              self.quadratic = common.fit_quadratic() 
35          else: 
36              # not used 
37              self.quadratic = None

38   
39   


40 -    def __call__(self, function, point, state):


41          """Assumes 1D parameter input""" 
42          res = [] 
43          for pval in self.samples: 
44              res.append(function.residual(np.array([pval]))[self.index]) 
45          if self.smooth: 
46              ixlo, ixhi = common.nearest_2n_indices(self.samples, 
47                                                     np.argmin(res), 2) 
48              smooth_res = common.smooth_pts(self.samples[ixlo:ixhi+1], 
49                                             res[ixlo:ixhi+1], 
50                                             self.quadratic) 
51              pmin = smooth_res.results.peak[0] 
52          else: 
53              pmin = self.samples[np.argmin(res)] 
54          state['direction'] = pmin - point 
55          return pmin

56
```

  


| Home | Trees | Indices | Help | PyDSTool |
| --- | --- | --- | --- | --- |

|  |  |
| --- | --- |
| Generated by Epydoc 3.0.1 on Fri May 4 15:24:18 2012 | http://epydoc.sourceforge.net |
